# In Vitro Evaluation of Aflatoxin B1 Detoxification by *Lactobacillus, Pediococcus,* and *Bacillus* Strains

**DOI:** 10.3390/toxins17080403

**Published:** 2025-08-11

**Authors:** Sarra Rafai, Ana Moreno, Alessandra Cimbalo, Pilar Vila-Donat, Lara Manyes, Giuseppe Meca

**Affiliations:** Biotech Agrifood Lab, Faculty of Pharmacy and Food Sciences, University of Valencia, Avda. Vicent Andrés Estellés s/n, 46100 Burjassot, València, Spain; sarra.rafai@uv.es (S.R.); ana.moreno@uv.es (A.M.); alessandra.cimbalo@uv.es (A.C.); lara.manyes@uv.es (L.M.); giuseppe.meca@uv.es (G.M.)

**Keywords:** biological detoxification, mycotoxins, lactic acid bacteria (LAB), in vitro digestion

## Abstract

Biologically based detoxification strategies are increasingly being explored as alternatives to conventional methods for the removal of toxic contaminants in food products. Among these, aflatoxin B1 (AFB1) is one of the most potent mycotoxins due to its high toxicity, genotoxicity, and persistence in the human body once ingested. In this study, the detoxification potential of bacterial strains belonging to the genera *Lactobacillus/Pediococcus* (n = 10) and *Bacillus* (n = 10) was evaluated using extracts from naturally contaminated corn flour. Detoxification was assessed after incubation for 12, 24, and 48 h in specific culture media. AFB1 quantification and metabolite profiling were performed at each time point using Quadrupole Time-of-Flight Mass Spectrometry (LC-QTOF-MS). The highest detoxification rates were observed with *Lactobacillus curvatus* 14 (*L. curvatus* 14) (41.1 ± 19.3%) and *Pediococcus pentosaceus* 4 (*P. pentosaceus* 4) (25.4 ± 11.3%) after 48 h, and *Bacillus firmus* 6 (*B. firmus* 6) (25.1 ± 12.9%) after 24 h. An in vitro digestion model was also applied to assess detoxification under gastrointestinal conditions. Results showed substantial AFB1 reduction at the colonic stage, reaching 72.26 ± 7.54% for *P. pentosaceus* 4 and 69.67 ± 9.70% for *L. curvatus* 14. These findings underscore the potential application of *Lactobacillus*, *Pediococcus*, and *Bacillus* strains in biological detoxification strategies to reduce dietary exposure to AFB1.

## 1. Introduction

Mycotoxins are toxic secondary metabolites produced predominantly by filamentous fungi such as *Aspergillus, Fusarium,* and *Penicillium*, which frequently contaminate agricultural commodities both before and after harvest. These compounds pose a serious threat to food safety and public health due to their hepatotoxic, nephrotoxic, immunosuppressive, genotoxic, and carcinogenic effects [1]. It is estimated that approximately 25% of global food crops are contaminated with mycotoxins each year, representing a significant risk for food security and international trade [2]. Moreover, climate change is exacerbating mycotoxin contamination by increasing the prevalence and geographic expansion of toxic fungi, such as *Aspergillus flavus* (*A. Flavus*), driven by rising temperatures and shifts in humidity and rainfall patterns [3]. Additionally, masked mycotoxins, which are conjugated derivatives often undetectable by standard analytical methods, represent a significant hidden threat. These modified forms can escape routine screening but may be enzymatically or chemically converted back into their toxic parent compounds during gastrointestinal digestion, potentially increasing overall exposure and risk [4].

Among the 400 identified mycotoxins, AFB1 stands out as the most toxic and prevalent. Produced mainly by *A. flavus* and *Aspergillus parasiticus*, AFB1 (C_17_H_12_O_6_) is classified as a Group 1 human carcinogen by the International Agency for Research on Cancer (IARC) due to its strong association with liver cancer [5]. Its chemical structure makes it highly stable and resistant to many conventional decontamination methods. AFB1 is only slightly soluble in water and demonstrates notable thermal stability, withstanding temperatures above 100 °C; some studies have reported partial degradation under high-temperature treatments. For instance, heating contaminated wheat at 150 °C and 200 °C has been shown to reduce AFB1 levels by approximately 50% and 90%, respectively [6]. In bakery matrices, baking conditions can also influence toxin reduction. Recent findings showed AFB1 reductions ranging from 47% to 89% during the baking of various traditional and industrial breads [7]. However, despite these decreases, complete detoxification is rarely achieved, and substantial levels of AFB1 may persist in the final product, thereby limiting the effectiveness of heat-based treatments such as pasteurization and cooking. Although certain physical or chemical conditions, like UV exposure or extreme pH, can reduce AFB1 levels, these are often impractical or damaging to food quality [8].

In this context, various mitigation strategies have been explored and classified into chemical, physical, and biological approaches. Chemical methods involve the application of acids, bases, oxidizing agents, aldehydes, or bisulfite gases to chemically alter the structure of mycotoxins and reduce their toxicity [9]. Physical methods aim to reduce mycotoxin content through non-chemical interventions such as sorting, milling, peeling, roasting, washing, drying, or irradiation [10]. In addition, inorganic adsorbents are widely used in animal feed to reduce AFB1 absorption [11]. However, despite their potential, chemical and physical approaches present notable limitations when applied to food matrices. These include safety concerns related to chemical residues, the potential loss of essential nutrients, adverse effects on organoleptic quality, and elevated operational costs [12]. In contrast, biological methods have emerged as a more environmentally friendly and food-compatible alternative [13]. These include the use of microorganisms such as bacteria, yeasts, and fungi or their enzymes to biotransform or adsorb mycotoxins into less toxic forms. Recent advances have highlighted the potential of probiotic bacteria for their ability to bind or degrade AFB1 in food and feed matrices [14]. Furthermore, fermentation using probiotics has been reported to significantly reduce AFB1 content in raw materials, suggesting a promising dual role in food preservation and detoxification [15].

Following this line, the present study aimed to investigate the detoxification potential of 20 bacterial strains, including species from the *Lactobacillus*/*Pediococcus* and *Bacillus* genera, against AFB1 present in contaminated maize flour extracts. To this end, each strain was evaluated through fermentation at three incubation times (12, 24, and 48 h), followed by an in vitro simulated digestion model to assess their detoxification efficiency under gastrointestinal conditions. In parallel, untargeted metabolomic analysis was used to characterize possible AFB1 degradation products. This approach allowed the identification of the most promising strains for future food-safe aflatoxin mitigation strategies.

Several studies have demonstrated the potential of *Lactobacillus* and *Bacillus* species in reducing AFB1 levels in vitro. For example, *Lactobacillus casei* and *L. plantarum* strains have shown significant binding and detoxification capacity under simulated intestinal conditions [16]. Similarly, *Bacillus subtilis* has been reported to effectively degrade AFB1, likely through enzymatic activity, highlighting its potential for use in feed and food detoxification strategies [17]. However, most of the existing research has focused on static fermentation or cell-free extracts, and relatively few studies have investigated the behavior of these strains under gastrointestinal-like conditions [18]. In contrast, our study evaluates both *Lactobacillus/Pediococcus* and *Bacillus* strains under controlled fermentation and digestion models, aiming to better identify candidate strains with robust activity across realistic physiological environments.

## 2. Results

### 2.1. AFB1 Quantification in Contaminated Flour and Pooled Extract

The analysis by LC-QTOF-MS revealed that the artificially contaminated flour contained 10.82 ± 0.06 (mg/kg) of AFB1. In the pooled liquid extract prepared from this flour, the AFB1 concentration reached 31.26 ± 3.26 (mg/L). This extract was subsequently used for detoxification and simulated digestion experiments.

### 2.2. Detoxification Potential of Bacterial Strains Toward AFB1

The detoxification potential of LAB against AFB1 was assessed at three fermentation time points: 12 h, 24 h, and 48 h. The initial AFB1 concentration in the control sample (without bacteria) was 244.15 ± 11.2 µg/L, and detoxification percentages were calculated relative to this value. Among the tested strains, only a few exhibited a marked reduction in AFB1 levels. *P. pentosaceus* 4 and *L. curvatus* 14 showed the highest detoxification capacities. *P. pentosaceus* 4 achieved a detoxification rate of 25.4% at 48 h, while *L. curvatus* 14 reached 41.1% at the same time point. In contrast, most other strains demonstrated limited efficacy, with detoxification rates remaining below 10% throughout the incubation period (Table 1).

Among the *Bacillus* strains, the initial AFB1 concentration in the control sample was 264 ± 4.08 µg/L, and all detoxification percentages were calculated relative to this value. *B. firmus* 6 showed the highest detoxification capacity, achieving a reduction of 25.1% at 24 h. *B. megaterium* 5 and *B. subtilis* 8 exhibited moderate detoxification rates of 14.3% and 11.7%, respectively, at 48 h. *B. siamnensis* 4 demonstrated consistent detoxification activity across the fermentation period, with rates ranging from 12.1% to 19.6%. On the other hand, strains such as *B. amyloliquefaciens* 20, *B. subtilis* 1, and *B. amyloliquefaciens* 7 did not exhibit any detoxification at the tested time points (Table 2).

### 2.3. Metabolite Identification Following AFB1 Detoxification

Metabolite analysis revealed that both LAB and *Bacillus* strains were capable of transforming AFB1 into various degradation products, with aflatoxicol being the most consistently detected metabolite across all strains and time points, at concentrations ranging from 31.00 to 55.40 µg/L. *L. curvatus* 14 and *B. siamensis* 4 exhibited the highest aflatoxicol levels, peaking at 47.45 µg/L and 55.40 µg/L, respectively, at 48 h and 24 h. In addition, *L. curvatus* 14 and *P. pentosaceus* 4 produced aflatoxin D1, while *P. pentosaceus* 29 was the only strain to generate aflatoxin Q1. Among the *Bacillus* strains, *B. siamensis* 4, *B. firmus* 6, and *B. velezensis* 9 showed a broader metabolic profile, including aflatoxin D1, P2, B2a, and Q1, suggesting multiple biotransformation pathways. In contrast, some strains, such as *B. licheniformis* 10 and *B. amyloliquefaciens* 20, primarily produced aflatoxicol, with no other detectable metabolites, possibly indicating more limited degradation mechanisms (Table 3 and Table 4).

### 2.4. AFB1 Binding Efficiency to Bacterial Cell Pellets

The adsorption capacity of AFB1 to bacterial pellets varied among the tested strains. Among the *Lactobacillus* and *Pediococcus* strains, *L. fermentum* 8 and *L. paracasei* 10 exhibited the highest adsorption rates, with 7.03 ± 1.79% and 7.95 ± 3.60%, respectively. *L. curvatus* 14 also showed a moderate adsorption of 5.01 ± 0.77%. In contrast, *L. plantarum* 5 showed the lowest value (1.18 ± 1.85%). Regarding *Bacillus* strains, *B. subtilis* 1 demonstrated the highest AFB1 adsorption percentage (10.52 ± 1.56%), followed by *B. megaterium* 5 (5.49 ± 2.11%) and *B. firmus* 6 (4.45 ± 1.64%). Other *Bacillus* strains showed limited adsorption capacity, with values generally below 4%. These results indicate strain-dependent variability in AFB1 binding, with certain strains showing promising adsorption potential (Table 5 and Table 6).

### 2.5. Sterile In Vitro Digestion

#### 2.5.1. AFB1 Detoxification Efficiency During Simulated In Vitro Digestion

**Figure 1:**
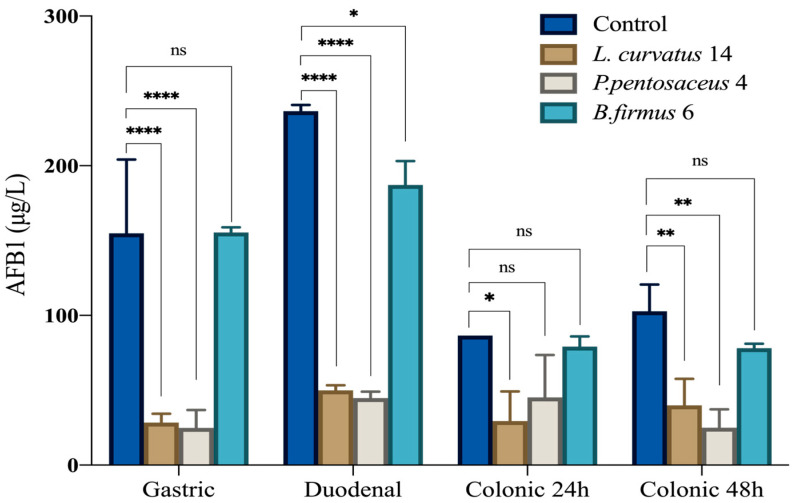
AFB1 concentration during in vitro digestion in the presence of selected bacterial strains compared to controls. The simulated digestion included four time points: gastric, duodenal, and colonic phases at 24 h and 48 h. Data are expressed as mean ± standard deviation (n = 2). Significant differences between groups are indicated by asterisks: * *p* < 0.05; ** *p* < 0.01; **** *p* < 0.0001. ns: not significant.

Based on the initial screening of AFB1 detoxification capabilities, three bacterial strains exhibiting distinct behaviors were selected for further investigation using the simulated digestion model: *L. curvatus* 14, *P. pentosaceus* 4, and *B. firmus* 6, which showed the highest detoxification levels. The simulated digestion model revealed variable detoxification capacities among the tested strains across the gastric, duodenal, and colonic phases. In the gastric phase, *L. curvatus* 14 and *P. pentosaceus* 4 achieved substantial reductions in AFB1 levels (81.6% and 83.9%, respectively), whereas *B. firmus* 6 showed no effect compared to the control. In the duodenal phase, similar trends were observed, with *L. curvatus* 14 and *P. pentosaceus* 4 reducing AFB1 by over 78%, while *B. firmus* 6 showed a moderate detoxification of 20.8%. In the colonic phase, a decrease in AFB1 concentration was also observed in the control group, dropping from 236.47 μg/L in the duodenal phase to 86.53 μg/L at 24 h and 90.12 μg/L at 48 h, possibly due to spontaneous and natural degradation. Nonetheless, *L. curvatus 14* and *P. pentosaceus* 4 maintained effective detoxifications (65.9% and 47.8% at 24 h), whereas *B. firmus* 6 exhibited limited reduction (8.5%). After 48 h, detoxification by *L. curvatus* 14 and *P. pentosaceus* 4 further improved to 69.7% and 72.3%, respectively, while *B. firmus* 6 remained less effective (13.3%) (Figure 1 and Table 7).

#### 2.5.2. Metabolite Profiling of AFB1 After Simulated Digestion

Aflatoxicol (Table 8) was detected exclusively in the control and *B. firmus* 6 samples throughout the digestion phases, with comparable concentrations ranging from 32.23 to 8.65 μg/L in the control and from 25.28 to 8.14 μg/L in *B. firmus* 6. In contrast, this metabolite was not detected in any samples treated with *L. curvatus* 14 or *P. pentosaceus* 4, suggesting their potential to inhibit the formation or accumulation of aflatoxicol during digestion.

## 3. Discussion

Our study provides new insights into the environmentally friendly detoxification of AFB1 using selected bacterial strains. Given AFB1’s extreme resistance to thermal and chemical treatments, even after intensive processing steps such as cooking or sterilization [19], biological approaches have emerged as safer and more sustainable alternatives [20]. In this context, we evaluated the detoxification potential of LAB and *Bacillus* strains through fermentation and simulated in vitro digestion. Our results highlight a strong detoxification performance by *L. curvatus* 14 and *P. pentosaceus* 4, particularly under gastrointestinal-like conditions, which underscores their potential as bio-based and eco-friendly solutions for AFB1 mitigation [20].

In the fermentation assay, detoxification patterns varied across strains and incubation durations. *L. curvatus* 14 and *P. pentosaceus* 4 exhibited the highest detoxification at 48 h, whereas *B. firmus* 6 showed peak activity at 24 h before declining. This temporal variability likely reflects strain-specific enzymatic stability or activity regulation. These findings are consistent with Escrivá et al. [21], who observed time-dependent detoxification increases in LAB across 2 to 72 h of fermentation. Additionally, Liu et al. [22] and Yang et al. [23] reported that optimal detoxification periods for microbial strains typically range from 12 to 48 h. The cell wall structure, particularly the presence of polysaccharides, plays a key role in toxin interaction and may explain inter-strain differences [24].

Importantly, our results revealed that the detoxification performances of *L. curvatus* 14, *P. pentosaceus* 4, and *B. firmus* 6 were comparable to those previously reported by Ondiek et al. [25] in food matrices. Furthermore, strains from the *Bacillus* genus, such as B. licheniformis CFR1, have also shown remarkable degradation efficiency in other studies, with over 90% AFB1 removal in field matrices [26], further supporting the relevance of bacilli in detoxification strategies.

Mechanistically, AFB1 detoxification by bacteria is generally attributed to two complementary processes: adsorption to the cell surface and enzymatic biotransformation [27]. In our fermentation assay, the relatively low recovery of AFB1 in the bacterial pellets suggested a limited contribution from adsorption. This aligns with the hypothesis that enzymatic degradation was the dominant mechanism. Metabolomic analysis confirmed this by detecting known AFB1 degradation products such as aflatoxicol, aflatoxin D1, and P2. Aflatoxicol is a reductive metabolite commonly observed in microbial systems, and its toxicity is significantly lower than that of parent AFB1 [19], supporting its relevance as a detoxification endpoint.

The in vitro digestion experiment was conducted to evaluate whether the selected strains could maintain their detoxification potential under gastrointestinal-like conditions. This approach aimed to mimic the complexity of the human intestinal tract and confirm the functional relevance of the bacterial strains beyond simple fermentation models. Both *L. curvatus* 14 and *P. pentosaceus* 4 demonstrated substantial and consistent detoxification activity throughout the gastric, duodenal, and colonic phases. In contrast, while *B. firmus* 6 showed promising results during fermentation, its efficacy dropped drastically during digestion, suggesting a lack of metabolic stability or adaptation in simulated intestinal conditions. These findings underline the superior physiological robustness of *L. curvatus* 14 and *P. pentosaceus* 4 and reinforce their potential use in food safety applications requiring gastrointestinal functionality. It is noteworthy that both the control and *B. firmus* groups exhibited a decrease in AFB1 concentration during the colonic phase, suggesting a natural reduction of the toxin, possibly mediated by microbial activity. This observation aligns with the findings of Sobral et al. [28], in which colonic fermentation by human microbiota led to a reduction in mycotoxin toxicity, as evidenced by the absence of NF-κB activation following fecal supernatant exposure. These results highlight the potential role of the gut microbiota in modulating AFB1 bioavailability and toxicity during the final stages of digestion.

These detoxification levels were considerably higher than those reported by Kabak and Ozbey [29], who observed bioaccessibility reductions between 18.1% and 35.6% using probiotic strains. Similarly, Zolfaghari et al. [30] demonstrated that *L. rhamnosus,* under simulated gastrointestinal conditions, achieved a maximum AFB1 reduction of 31.14%. Our data therefore confirms the superior performance of *L. curvatus* 14 and P. *pentosaceus* 4 under physiologically relevant conditions, nearly doubling the detoxification efficiency previously reported in comparable in vitro models.

A particularly novel finding of our study is the complete absence of detectable AFB1 degradation products following digestion with *L. curvatus* 14 and *P. pentosaceus* 4. This contrasted with the results observed for the control and *B. firmus* 6, where aflatoxicol was present. The absence of detectable metabolites could be interpreted as either irreversible binding, as described by Liu et al. [24], or as evidence of complete enzymatic degradation into compounds not recognized among currently described AFB1 metabolites. The absence of aflatoxicol, which has been detected in human and animal urine and feces [31], reinforces the hypothesis of a complete detoxification process mediated by the two selected strains. This suggests that degradation products may have been rapidly further metabolized into non-identifiable compounds. Thus, the endpoint of detoxification might not correspond to classical known metabolites, indicating that degradation under probiotic influence may follow alternative metabolic pathways. To our knowledge, no previous study has reported a full in vitro digestion model where no known AFB1 metabolites were detected after treatment, highlighting the uniqueness and potential strength of our system.

Altogether, our findings support the application of *L. curvatus* 14 and *P. pentosaceus* 4 as promising candidates for biological decontamination. Their high detoxification capacity, consistent behavior in fermentation and in vitro digestion, and the apparent safety of their metabolic outcomes underline their value in food protection. These results reinforce the role of microbial strategies as efficient, natural, and sustainable solutions for mycotoxin mitigation.

## 4. Conclusions

In conclusion, this study highlights the promising potential of selected bacterial strains, notably *L. curvatus* 14 and *P. pentosaceus* 4, for effective detoxification of AFB1 in contaminated maize flour. These strains demonstrated strong and sustained detoxification capacity across simulated digestion phases. Importantly, metabolomic analyses did not reveal the formation of toxic metabolites during any of the experiments, supporting the safety of these biological detoxification processes. Overall, the results reinforce the value of the selected bacterial strains as eco-friendly and food-compatible agents for mycotoxin mitigation, paving the way for further research and practical applications to improve food safety and public health.

Nevertheless, further research is warranted to evaluate the toxicity and potential biological effects of the AFB1 degradation products formed during bacterial detoxification. A comprehensive toxicological assessment is essential to confirm that these transformation products do not pose new health risks and to validate the safety of implementing these microbial strains in food and feed decontamination strategies.

## 5. Materials and Methods

### 5.1. Chemicals and Reagents

AFB1 standard (purity > 99%) was obtained from Sigma-Aldrich (St. Louis, MO, USA). All stock solutions (1000 µg/mL) were prepared by dissolving 1 mg of AFB1 in 1 mL of pure methanol. These stock solutions were subsequently diluted with methanol to obtain the desired working standard solutions, which were stored at −20 °C until use. Potassium chloride (KCl), potassium thiocyanate (KSCN), sodium dihydrogen phosphate (NaH_2_PO_4_), sodium sulfate (Na_2_SO_4_), sodium chloride (NaCl), sodium hydrogen carbonate (NaHCO_3_), urea (CO(NH_2_)_2_), α-amylase (930 U/mg, A3403), hydrochloric acid (HCl), sodium hydroxide (NaOH), formic acid (HCOOH), pepsin A (674 U/mg, P7000), pancreatin (762 U/mg, P1750), bile salts (B8631), and phosphate-buffered saline (PBS, pH 7.5) were purchased from Sigma-Aldrich (Madrid, Spain). Methanol and LC/MS-grade acetonitrile (≥99.9%) were supplied by Fisher Scientific (Madrid, Spain). Deionized water (resistivity ≥ 18 MΩ·cm) was obtained from a Milli-Q water purification system (Millipore, Bedford, MA, USA). De Man, Rogosa, and Sharpe (MRS) broth and Nutrient Broth (NB) were obtained from Liofilchem Bacteriology Products (Roseto degli Abruzzi, Italy). Formic acid (≥98%) was obtained from Sigma Aldrich (St. Louis, MO, USA).

### 5.2. Fungal Contamination and AFB1 Production

To produce AFB1-contaminated flour, *Aspergillus flavus* (ITEM 8111) was obtained from the Microbial Culture Collection of the Institute of Sciences of Food Production (ISPA, Bari, Italy) and used to naturally contaminate sterilized maize. The fungal strain was grown on potato dextrose agar (PDA), and spores were harvested by adding sterile peptone water containing 0.1% Tween 80 (Thermo Fisher Scientific, Waltham, MA, USA) to the agar surface. The resulting spore–mycelium suspension was adjusted to a known concentration and used to inoculate approximately 400–450 g of sterilized maize placed in 1 L glass jars that had been previously autoclaved. Each jar received 15–20 mL of the fungal suspension under aseptic conditions. The jars were incubated at room temperature in the dark for four weeks to allow natural fungal colonization and mycotoxin production. After incubation, the contaminated maize was autoclaved to eliminate viable fungal growth, dried, and then finely ground into flour to ensure homogeneity. The AFB1 content in the contaminated maize flour was quantified using LC-QTOF-MS, detailed in Section 5.6.1.

### 5.3. Bacterial Strains and Culture Conditions

A total of 20 bacterial strains were evaluated in this study, including 10 strains of *Lactobacillus/Pediococcus and* 10 strains of *Bacillus*. All strains were obtained from the Spanish Type Culture Collection (CECT), located at the Science Park of the University of Valencia (Paterna, València, Spain), and preserved at −80 °C in 25% glycerol until use. The LAB included: *L. paracasei* 1, *L. paracasei* 10, *P. acidilactici* 2, *P. acidilactici* 3, *P. pentosaceus* 4, *P. pentosaceus* 29, *L. plantarum* 5, *L. plantarum* 6, *L. fermentum* 8, and *L. curvatus* 14.

The Bacillus strains included: *B. subtilis* 1, *B. amyloliquefaciens* 2, *B. siamensis* 4, *B. megaterium 5*, *B. firmus* 6, *B. amyloliquefaciens* 7, *B. subtilis* 8, *B. velezensis* 9, *B. licheniformis* 10, and *B. amyloliquefaciens* 20.

For activation before their use in detoxification assays, bacterial cultures were incubated overnight. *Lactobacillus* strains were cultivated at 37 °C in MRS broth under anaerobic conditions, whereas *Bacillus* strains were grown at 30 °C in NB under aerobic conditions.

### 5.4. In Vitro Detoxification Assay

For each bacterial strain, a total volume of 5 mL of fresh culture medium was prepared using MRS for *Lactobacillus* strains and NB for *Bacillus* strains. Each medium was inoculated with 1% (*v*/*v*) of an overnight bacterial suspension. Cultures were supplemented with 20 µL of naturally contaminated maize flour extract, corresponding to an approximate AFB1 concentration of 200 µg/L. Incubations were performed at 37 °C for *Lactobacillus* and 30 °C for *Bacillus* strains. Samples were collected at 12, 24, and 48 h. At each time point, 200 µL of culture was withdrawn, diluted with 800 µL of methanol, and filtered through a 0.22 µm membrane filter prior to LC-QTOF-MS analysis. Controls consisting of culture media with AFB1 extract but without bacterial inoculation were included for comparison. All samples were processed and analyzed in duplicate to evaluate AFB1 detoxification efficiency. The percentage of detoxification was then directly calculated using the formula provided in Equation (1):

(1) $$Detoxification\;\left(\%\right)=100-\frac{sample\;concentration\;\left(µg/L)\;\right)\times 100}{Control\;Concentration\;(µg/L)\;\;}$$

### 5.5. Extraction of AFB1

#### 5.5.1. Extraction from Contaminated Flour

After incubation, the artificially contaminated maize flour was dried, finely ground, and thoroughly homogenized. AFB1 was extracted using a liquid-solid extraction method. Briefly, 5 g of contaminated flour was mixed with 25 mL of absolute methanol in a sterile container. The mixture was homogenized for 5 min using an Ultra-Turrax homogenizer (model T 18 digital ULTRA-TURRAX^®^, Staufen, Germany) and then centrifuged at 4000 rpm for 5 min (Centrifuge 5810R, Eppendorf, Hamburg, Germany). The supernatant was carefully collected, evaporated to dryness under controlled temperature conditions, and reconstituted in 1 mL of methanol. Extracts were transferred to amber vials and stored at −20 °C until LC-QTOF-MS analysis.

To ensure sufficient toxin concentration for detoxification and simulated digestion experiments, a pooled extract was prepared by combining individual extracts to achieve a higher AFB1 concentration. This pooled extract was used for experimental assays.

#### 5.5.2. Extraction from Bacterial Pellets

To evaluate the extent of AFB1 binding or intracellular retention by bacterial cells, cultures were centrifuged at 4000 rpm for 5 min, and the resulting pellets were washed three consecutive times with approximately 3 mL of sterile PBS to remove extracellular AFB1 residues. After washing, the bacterial pellets were extracted with 2 mL of absolute methanol and subjected to sonication for 20 min to facilitate the release of bound or internalized AFB1. The mixture was then centrifuged at 4000 rpm for 5 min, and the supernatant was collected. This methanolic extraction step was repeated two additional times under the same conditions to maximize AFB1 recovery. The combined supernatants (total volume: 6 mL) were evaporated to dryness and reconstituted in 1 mL of methanol. The final extracts were transferred into amber vials and stored at −20 °C until LC-QTOF-MS analysis. The percentage of adsorption was calculated using the formula provided in Equation (2):

(2) $$Adsorption\left(\%\right)=\frac{\left(AFB1\;in\;pellet\left(\mathrm{ng}\right)\times 100\right)}{AFB1\;initially\;added(\mathrm{ng})}$$

### 5.6. LC-QTOF-MS Conditions

#### 5.6.1. AFB1 Quantification

AFB1 quantification was carried out using LC-QTOF-MS. Chromatographic separation was performed using an Agilent 1200 Infinity Series LC system (Agilent Technologies, Santa Clara, CA, USA) coupled to an Agilent 6540 UHD Accurate-Mass QTOF mass spectrometer equipped with a Dual Jet Stream electrospray ionization source (Dual AJS ESI) operating in positive ionization mode. The separation was achieved using a Gemini C18 column (50 mm × 2 mm, 110 Å, 3 µm particle size; Phenomenex, Palo Alto, CA, USA) maintained at 40 °C. The mobile phase consisted of water with 0.1% formic acid (solvent A) and acetonitrile with 0.1% formic acid (solvent B). The gradient elution was programmed as follows: 90% A at 0 min, 70% A at 1.5 min, 30% A at 10.0 min, 5% A at 11.0 min, held at 5% A until 13.0 min, and returned to 90% A at 14.0 min. The flow rate was set to 0.5 mL/min, and the injection volume was 2 µL. Calibration curves were constructed using 9 concentration levels ranging from 3.95 ng/mL to 1000 ng/mL, covering a broad dynamic range suitable for sample analysis. The calibration showed excellent linearity, with a regression coefficient (r^2^) of 0.9972.

The QTOF-MS operated in targeted MS/MS mode with the following parameters: gas temperature 330 °C, drying gas flow 10 L/min, sheath gas temperature 350 °C, sheath gas flow 11 L/min, nebulizer pressure 45 psi, capillary voltage 3500 V, nozzle voltage 500 V, fragmentor 160 V, skimmer 30 V, and Octopole RF Peak at 750 V. The MS scan range was *m*/*z* 100–1700 at 2 spectra/sec, and the MS/MS scan range was *m*/*z* 50–1700 at 8 spectra/sec. Collision energies of 10, 20, and 40 eV were applied for fragmentation. Results were expressed in µg/kg for flour samples and in µg/L for culture supernatants. Data acquisition and processing were performed using MassHunter Qualitative Analysis Software Version B.08.00 (Agilent Technologies, Santa Clara, CA, USA).

#### 5.6.2. Metabolite Detection LC-QTOF-MS

To investigate potential biotransformation or degradation products of AFB1, an untargeted metabolomics analysis was performed using LC-QTOF-MS. The analyses were conducted under the same chromatographic and spectrometric conditions previously described in Section 5.6.1. Both positive and negative ionization modes were applied to ensure comprehensive metabolite detection.

The gradient was programmed as follows: 10% A/90% B from 7.00 to 9.00 min, then shifted to 98% A/2% B from 10.00 to 15.00 min. The total runtime was 15 min, and the injection volume was 10 µL. Full-scan MS data were acquired in the m/z range 50–1500 at a scan rate of 2 spectra/sec. Source parameters were set as follows: gas temperature 330 °C, drying gas flow 10 L/min, sheath gas temperature 350 °C, sheath gas flow 11 L/min, nebulizer pressure 45 psi, capillary voltage 3500 V, nozzle voltage 250 V, fragmentor 160 V, skimmer 30 V, and Octopole RF Peak 750 V. Data processing and compound identification were performed using MassHunter Personal Compound Database and Library (PCDL) Manager Software, version B.08.00 (Agilent Technologies), by screening against internal libraries and curated literature databases of known or suspected AFB1 degradation products. Identification was further supported by accurate mass, isotopic pattern, and fragment ion analysis. Only compounds with a library match score greater than 80% were considered.

### 5.7. Sterile In Vitro Digestion

An in vitro digestion model was developed to simulate the human gastrointestinal tract, including oral, gastric, duodenal, and colonic phases, to assess AFB1 stability and the detoxification potential of selected bacterial strains. Each digestion was performed in duplicate, with a final volume of 100 mL per sample, composed of 6 mL of freshly prepared artificial saliva, 357 µL of pooled AFB1 extract (corresponding to a final concentration of approximately 150 µg/L), 10% (*v*/*v*) of a bacterial suspension for treated samples (not added in controls), and sterile Milli-Q water to reach the final volume. Artificial saliva was prepared by mixing inorganic components (1 mL each of KCl 89.6 g/L, KSCN 20 g/L, NaH_2_PO_4_ 8.8 g/L, Na_2_SO_4_ 57 g/L, 0.17 mL of NaCl 175.3 g/L, and 2 mL of NaHCO_3_ 84.7 g/L) with 43.8 mL of distilled water. The pH was adjusted to 6.8 ± 0.2, and 29 mg of α-amylase and 2.5 mg of mucin were reconstituted to complete the artificial saliva. After mixing the saliva with the rest of the digestion components, the samples were transferred to sterile, darkened Erlenmeyer flasks covered with Parafilm and aluminum foil. The gastric phase was initiated by adjusting the pH to 2.0 using 6 N HCl and adding 0.5 mL of pepsin solution (1 g in 25 mL of 0.1 N sterile HCl). Samples were incubated at 37 °C for 2 h under agitation (100 rpm). The duodenal phase followed, with pH adjustment to 6.5 using 1 N NaHCO_3_, then addition of 1.25 mL of a freshly prepared bile salts–pancreatin solution (0.1 g pancreatin and 0.625 g bile salts in 25 mL of 0.1 N sterile NaHCO_3_), and incubation at 37 °C for another 2 h with agitation. At the end of the intestinal digestion, the pH was adjusted to 7.2 using 0.5 N NaOH, and the contents were transferred into sterile urine collection containers for the colonic phase. Colonic fermentation was simulated under anaerobic conditions using an Anaerocult A system: containers were placed in hermetically sealed jars containing a CO_2_-generating strip moistened with sterile water, then sealed with Parafilm. Samples were incubated at 37 °C for 48 h. Samples were collected after the gastric, duodenal, and colonic phases at 24 h and 48 h for LC-QTOF-MS analysis.

### 5.8. Statistical Analysis

All results are expressed as means ± standard deviation (SD). Statistical analysis and graphical representations were carried out using GraphPad Prism version 10.1.1 for Macintosh GraphPad Software, Boston, MA, USA (www.graphpad.com, accessed on 1 May 2025). Differences between groups were assessed using two-way analysis of variance (ANOVA), and a *p*-value ≤ 0.05 was considered statistically significant.

## Figures and Tables

**Table 1:** AFB1 concentrations (μg/L) and detoxification percentages (mean ± SD) by *Lactobacillus* and *Pediococcus* strains at different fermentation times (12 h, 24 h, and 48 h).

Fermentation Period	12 h Fermentation		24 h Fermentation		48 h Fermentation	
Bacterial Strain	AFB1 μg/L	% Detox	AFB1 μg/L	% Detox	AFB1 μg/L	% Detox
*L. paracasei* 1	222.5 ± 19.4	8.9 ± 7.9	212.5 ± 20.8	0.0	223.5 ± 10.2	3.9 ± 4
*L. paracasei* 10	252.9 ± 11.6	0.0	226.1 ± 6.1	0.0	236 ± 9.1	0.0
*P. acidilactici* 2	230.6 ± 54.8	5.6 ± 22.4	206.1 ± 6.0	3 ± 2	211 ± 23.6	9.3 ± 8.1
*P. acidilactici* 3	255.8 ± 6.9	0.0	260.9 ± 9.5	0.0	220 ± 30.4	5.9 ± 7.8
*P. pentosaceus* 4	189.2 ± 28.8	22.5 ± 11.8	225.4 ± 36.2	0.0	173.5 ± 29.8	25.4 ± 11.3
*P. pentosaceus* 29	239.4 ± 4.9	1.9 ± 2.0	215.4 ± 15.5	0.0	224.5 ± 8.5	3.9 ± 6.6
*L.plantarum* 5	241.8 ± 8.3	1.0 ± 3.4	244.7 ± 6.1	0.0	227 ± 6.4	2.4 ± 2.7
*L.plantarum* 6	267 ± 5.0	0.0	266.5 ± 5.0	0.0	230 ± 5.9	1.1 ± 3.1
*L. fermentum* 8	245.6 ± 23.3	0.0	249.4 ± 2.7	0.0	231 ± 13.8	0.6 ± 1.9
*L. curvatus* 14	251 ± 7.8	0.0	228.6 ± 1.3	0.0	137 ± 34.7	41.1 ± 19.3

%Detox = Detoxification of AFB1.

**Table 2:** AFB1 concentrations (μg/L) and detoxification percentages (mean ± SD) by *Bacillus* strains at different fermentation times (12 h, 24 h, and 48 h).

Fermentation Period	12 h Fermentation		24 h Fermentation		48 h Fermentation	
Bacterial Strain	AFB1 μg/L	% Detox	AFB1 μg/L	% Detox	AFB1 μg/L	% Detox
*B. subtilis* 1	281.2 ± 13.6	0.0	264 ± 6.5	0.0	253 ± 21.2	3.2 ± 8.1
*B. amyloliquefaciens* 2	250.6 ± 35.7	6.6 ± 13.2	237.2 ± 12.7	10.1± 4.7	242 ± 45.3	7.5 ± 17.3
*B. siamensis* 4	215.7 ± 38.2	19.6 ± 15.1	229.3 ± 35	13.2 ± 2.1	230 ± 35.4	12 ± 13.5
*B. megaterium* 5	250.75 ± 0.8	6.6 ± 0.2	253 ± 23.3	4.8± 5.1	224 ± 32.5	14.3 ± 12.4
*B. firmus* 6	273.3 ± 54.6	0.0	197.6 ± 44.2	25.1± 12.9	258.5 ± 2.1	1.1 ± 0.8
*B. amyloliquefaciens* 7	278.9 ± 13.3	0.0	238.4 ± 37.1	9.7± 4.7	272 ± 17	0.0
*B. subtilis* 8	271.2 ± 71.9	0.0	240.6 ± 47	8.9± 4.1	231 ± 9.9	11.7 ± 3.8
*B. velezensis* 9	255.3 ± 37.5	4.9 ± 10	234.3 ± 5.4	11.2± 1.1	250.5 ± 7.8	4.2 ± 3
*B. licheniformis* 10	242.1 ± 1.9	9.8 ± 0.1	250.4 ± 13.2	5.2± 3.3	262 ± 17	0.0
*B. amyloliquefaciens* 20	285.9 ± 34.9	0.0	263.9 ± 21.6	0.1± 1.1	269 ± 22.6	0.0

%Detox = Detoxification of AFB1.

**Table 3:** Metabolic profiling of AFB1 degradation products detected after fermentation with *Lactobacillus* and *Pediococcus* strains at 12 h, 24 h, and 48 h.

Bacteria Strain	Produced Metabolites	12 h Fermentation (μg/L)	24 h Fermentation (μg/L)	48 h Fermentation (μg/L)
*L. paracasei* 1	Aflatoxicol	33.44	33.07	33.80
	Aflatoxin P2	8.15	ND	ND
	Aflatoxin D1	ND	2.37	1.91
*L. paracasei* 10	Aflatoxicol	38.70	38.55	37.30
	Aflatoxin P2	ND	ND	7.29
	Aflatoxin P1	4.13	ND	ND
*P. acidilactici* 2	Aflatoxicol	40.67	34.33	53.92
	Aflatoxin B2a	17.89	ND	ND
*P. acidilactici* 3	Aflatoxicol	39.74	39.15	37.59
	Aflatoxin P2	10.25	ND	ND
*P. pentosaceus* 4	Aflatoxicol	31.00	33.22	33.48
	Aflatoxin D1	ND	4.03	10.90
*P. pentosaceus* 29	Aflatoxicol	42.22	31.18	38.18
	Aflatoxin P2	ND	8.34	ND
	Aflatoxin Q1	ND	5.23	ND
*L.plantarum* 5	Aflatoxicol	38.51	37.14	35.45
	Aflatoxin D1	2.90	ND	ND
*L.plantarum* 6	Aflatoxicol	40.57	39.07	41.55
	Aflatoxin P2	ND	7.97	11.25
	Aflatoxin D1	ND	1.70	6.95
*L. fermentum* 8	Aflatoxicol	39.60	34.62	37.72
	Aflatoxin D1	ND	ND	1.80
	Aflatoxin P2	9.15	9.87	ND
*L. curvatus* 14	Aflatoxicol	45.14	44.37	47.45
	Aflatoxin D1	2.10	30.13	ND

ND = Not detected.

**Table 4:** Metabolic profiling of AFB1 degradation products detected after fermentation with *Bacillus* strains at 12 h, 24 h, and 48 h.

Bacteria Strain	Produced Metabolites	12 h Fermentation (μg/L)	24 h Fermentation (μg/L)	48 h Fermentation (μg/L)
*B. subtilis* 1	Aflatoxicol	40.47	40.80	39.67
	Aflatoxin D1	ND	2.45	ND
	Aflatoxin P2	ND	9.44	ND
*B. amyloliquefaciens* 2	Aflatoxicol	43.24	35.32	41.36
	Aflatoxin P2	7.56	ND	14.84
*B. siamensis* 4	Aflatoxicol	40.94	55.40	49.79
	Aflatoxin D1	ND	22.80	ND
	Aflatoxin B2a	ND	2.16	ND
	Aflatoxin P2	ND	9.98	ND
	Aflatoxin Q1	3.77	ND	ND
*B. megaterium* 5	Aflatoxicol	41.17	39.34	34.41
	Aflatoxin D1	ND	3.28	ND
	Aflatoxin P2	ND	6.28	ND
*B. firmus* 6	Aflatoxicol	39.06	31.80	37.52
	Aflatoxin D1	ND	4.49	ND
	Aflatoxin P2	7.55	11.28	ND
	Aflatoxin B2a	ND	ND	9.16
*B. amyloliquefaciens* 7	Aflatoxicol	39.67	38.03	41.98
	Aflatoxin P1	3.64	ND	ND
	Aflatoxin B2a	ND	4.01	ND
*B. subtilis* 8	Aflatoxicol	40.03	46.52	42.55
*B. velezensis* 9	Aflatoxicol	37.79	36.97	36.33
	Aflatoxin B2a	ND	9.18	ND
	Aflatoxin P2	ND	6.72	9.56
	Aflatoxin D1	3.83	2.69	3.36
*B. licheniformis* 10	Aflatoxicol	35.21	36.92	43.22

ND = Not detected.

**Table 5:** Adsorption percentages (%) (mean ± SD) of AFB1 by *Lactobacillus* and *Pediococcus* strains following in vitro incubation.

Bacterial Strain	Pellet Weight (g)	Quantity AFB1 Pellet (ng)	% Adsorption Pellet
*L. paracasei* 1	0.04	57.25 ± 19.95	4.69 ± 1.63
*L. paracasei* 10	0.04	97.06 ± 14.93	7.95 ± 3.60
*P. acidilactici* 2	0.08	53.92 ± 12.13	4.42 ± 1.50
*P. acidilactici* 3	0.07	46.26 ± 18.37	3.79 ± 1.74
*P. pentosaceus* 4	0.05	34.54 ± 21.32	2.83 ± 1.09
*P. pentosaceus* 29	0.05	57.65 ± 13.35	4.72 ± 1.71
*L.plantarum* 5	0.05	14.40 ± 10.80	1.18 ± 1.85
*L.plantarum* 6	0.04	83.15 ± 22.12	6.81 ± 3.69
*L. fermentum* 8	0.05	85.86 ± 12.85	7.03 ± 1.79
*L. curvatus* 14	0.05	61.12 ± 9.46	5.01 ± 0.77

**Table 6:** Adsorption percentages (%) (mean ± SD) of AFB1 by *Bacillus* strains following in vitro incubation.

Bacterial Strain	Pellet Weight (g)	Quantity AFB1 Pellet (ng)	% Adsorption Pellet
*B. subtilis* 1	0.07	141.18 ± 20.98	10.52 ± 1.56
*B. amyloliquefaciens* 2	0.05	49.04 ± 13.79	3.65 ± 2.51
*B. siamensis* 4	0.03	48.79 ± 1.51	3.63 ± 0.11
*B. megaterium* 5	0.04	73.71 ± 8.93	5.49 ± 2.11
*B. firmus* 6	0.03	59.73 ± 12.13	4.45 ± 1.64
*B. amyloliquefaciens* 7	0.05	19.40 ± 18.04	1.45 ± 1.34
*B. subtilis* 8	0.04	22.62 ± 13.51	1.69 ± 1.00
*B. velezensis* 9	0.06	56.75 ± 14.65	4.23 ± 3.41
*B. licheniformis* 10	0.06	21.86 ± 17.37	1.63 ± 1.29
*B. amyloliquefaciens* 20	0.05	30.72 ± 2.03	2.29 ± 0.12

**Table 7:** AFB1 concentration (μg/L) and detoxification percentage (mean ± SD) during in vitro digestion.

Digestive Phase	Sample	AFB1 (μg/L)	% Detoxification
Gastric	Control	154.86 ± 49.23	-
	*L. curvatus* 14	28.48 ± 5.86 ****	81.61 ± 3.78
	*P. pentosaceus* 4	24.87 ± 11.93 ****	83.93 ± 7.70
	*B. firmus* 6	155.50 ± 3.29	−0.41 ± 2.12
Duodenal	Control	236.47 ± 4.16	-
	*L. curvatus* 14	49.93 ± 3.44 ****	78.88 ± 1.45
	*P. pentosaceus* 4	44.74 ± 4.30 ****	81.07 ± 1.81
	*B. firmus* 6	187.21 ± 15.95	20.83 ± 6.83
Colonic 24 h	Control	86.53 ± 8.11	-
	*L. curvatus* 14	29.45 ± 9.76 *	65.96 ± 12.87
	*P. pentosaceus* 4	45.21 ± 8.36	47.75 ± 9.78
	*B. firmus* 6	79.12 ± 6.87	8.55 ± 7.94
Colonic 48 h	Control	90.12 ± 17.76	-
	*L. curvatus* 14	27.33 ± 8.76 **	69.67 ± 9.70
	*P. pentosaceus* 4	24.99 ± 6.20 **	72.26 ± 7.54
	*B. firmus* 6	78.15 ± 3.00	13.28 ± 3.33

* *p* < 0.05; ** *p* < 0.01; **** *p* < 0.0001, significantly different from control.

**Table 8:** Aflatoxicol concentration (μg/L) detected after in vitro digestion at four time points: gastric, duodenal, and colonic phases at 24 h and 48 h.

Digestion Phase	Sample	Aflatoxicol (μg/L)
Gastric	Control	32.23 ± 0.39
	*L. curvatus* 14	ND
	*P. pentosaceus* 4	ND
	*B. firmus* 6	25.28 ± 3,95
Duodenal	Control	39.17 ± 0.07
	*L. curvatus* 14	ND
	*P. pentosaceus* 4	ND
	*B. firmus* 6	29.79 ± 2.83
Colonic 24 h	Control	9.78 ± 4.15
	*L. curvatus* 14	ND
	*P. pentosaceus* 4	ND
	*B. firmus* 6	8.41 ± 0.16
Colonic 48 h	Control	8.65 ± 0.30
	*L. curvatus* 14	ND
	*P. pentosaceus* 4	ND
	*B. firmus* 6	8.14 ± 1.01

ND = Not detected.

## Data Availability

The original contributions presented in this study are included in the article. Further inquiries can be directed to the corresponding author.

## Abbreviations

The following abbreviations are used in this manuscript:

*A. flavus*	*Aspergillus flavus*
AFB1	Aflatoxin B1
*B. amyloliquefaciens* 2	*Bacillus amyloliquefaciens* 2
*B. amyloliquefaciens* 20	*Bacillus amyloliquefaciens* 20
*B. amyloliquefaciens* 7	*Bacillus amyloliquefaciens* 7
*B. firmus* 6	*Bacillus firmus* 6
*B. licheniformis* 10	*Bacillus licheniformis* 10
*B. megaterium* 5	*Bacillus megaterium* 5
*B. siamensis* 4	*Bacillus siamensis* 4
*B. subtilis* 1	*Bacillus subtilis* 1
*B. subtilis* 8	*Bacillus subtilis* 8
*B. velezensis* 9	*Bacillus velezensis* 9
IARC	International Agency for Research on Cancer
*L. curvatus* 14	*Lactobacillus curvatus* 14
*L. fermentum* 8	*Lactobacillus fermentum* 8
*L. paracasei* 1	*Lactobacillus paracasei* 1
*L. paracasei* 10	*Lactobacillus paracasei* 10
*L. plantarum* 5	*Lactobacillus plantarum* 5
*L. plantarum* 6	*Lactobacillus plantarum* 6
LAB	Lactic Acid Bacteria
LC-QTOF-MS	Quadrupole Time-of-Flight Mass Spectrometry
MRS	De Man, Rogosa and Sharpe
NB	Nutrient Broth
*P. acidilactici* 2	*Pediococcus acidilactici* 2
*P. acidilactici* 3	*Pediococcus acidilactici* 3
*P. pentosaceus* 29	*Pediococcus pentosaceus* 29
*P. pentosaceus* 4	*Pediococcus pentosaceus* 4
